# Self-expandable duodenal metal stent placement for the palliation of gastric outlet obstruction over the past 20 years

**DOI:** 10.1055/a-1838-5642

**Published:** 2022-06-23

**Authors:** Agnes N. Reijm, Pauline A. Zellenrath, Ruben D. van der Bogt, Lydi M. J. W. van Driel, Peter D. Siersema, Marco J. Bruno, Manon C. W. Spaander

**Affiliations:** 1 Department of Gastroenterology and Hepatology, Erasmus MC University Medical Center, Rotterdam, The Netherlands; 2 Department of Gastroenterology and Hepatology, Radboud University Medical Center, Nijmegen, The Netherlands

## Abstract

**Introduction** Duodenal stent placement is a palliative option for management of malignant gastric outlet obstruction (GOO). In the last 20 years, management of gastrointestinal cancers has considerably changed. It is unknown if these changes have affected clinical outcome of duodenal stent placement.

**Methods** Retrospective cohort study conducted in a tertiary referral center. Patients who underwent duodenal stent placement for GOO-symptoms due to a malignant stricture were included. Primary outcome was GOO-symptom free survival. Secondary outcomes included stent-related adverse event rates. Potential explanatory parameters such as period of stent placement (1998–2009 vs 2010–2019), prior treatments, peritoneal deposits, and stricture length were evaluated using multivariable Cox regression analysis.

**Results** A total of 147 patients (62 % male; median age 64 years) were included. After a median of 28 days after stent placement, 82 patients (57 %) had recurrent GOO-symptoms. GOO-symptom free survival was significantly lower in 2010–2019 (P < 0.01). Time period was the only independent predictor for reduced GOO-symptom free survival (HR 1.76, P < 0.01). Stent-related adverse event rates increased over time (1998–2009: 31 % vs 2010–2019: 37 %). Prior treatment with chemotherapy and/or radiotherapy was significantly associated with an increased risk of adverse events (OR 2.53, P = 0.02).

**Conclusions** Clinical outcome of duodenal stent placement did not improve over time. The decreased GOO-symptom free survival and increased adverse event rate in more recent years are probably related to the chemo- and/or radiotherapy treatment provided prior to duodenal stent placement.

## Introduction

Endoscopic treatment has emerged as a less invasive alternative to surgical gastrojejunostomy in the palliation of gastric outlet obstruction (GOO) symptoms 1 2 . Available endoscopic options include the placement of a self-expandable metal stent (SEMS; hereinafter referred to as the “stent”) and the more recently introduced endoscopic ultrasound (EUS)-guided gastrojejunostomy 3 4 . As EUS-guided gastrojejunostomy requires expert interventional EUS skills and is not feasible in all patients, endoscopic stent placement remains the mainstay of palliative endoscopic treatment of GOO symptoms.

Different stent designs are available for the palliation of GOO symptoms. Previous studies have shown uncovered duodenal stent placement to be highly successful in the palliation of GOO symptoms, with a technical success rate of 100 % and a clinical success rate of 86 % 3 . In the long term, however, a considerable number of patients experience a recurrence of symptoms requiring additional interventions. Moreover, adverse events (AEs) are common. Little is known about the parameters that affect GOO symptom recurrence and AE rates after duodenal stent placement, as only a few studies have investigated the impact of patient- and disease-specific characteristics on stent-related outcomes 5 6 7 8 . Furthermore, none of these studies have investigated changes over time in stent-related outcomes, while ongoing technical developments in stent design and changes in the management strategies for the underlying malignant disease may have affected clinical outcomes. Therefore, in this study we evaluated the clinical outcomes of duodenal stent placement for the palliation of GOO symptoms over a period of 20 years and aimed to assess trends over time with regard to recurrent GOO symptoms and the number of AEs.

## Methods

A retrospective study was performed of patients who underwent duodenal stent placement for palliation of GOO symptoms between January 1998 and November 2019 in the Erasmus MC University Medical Center in the Netherlands, which serves as a tertiary referral center. Potentially eligible patients were identified using a text search in the statistic module of ENDOBASE (Olympus, Tokyo, Japan), an endoscopy documentation system, to find all endoscopy reports in which duodenal stent placement was mentioned.

Before the onset of data collection, the study protocol was approved by the Medical Research Ethics Committee of the Erasmus MC University Medical Center (MEC-2019–0251).

### Patient selection

Patients were eligible if they had GOO symptoms due to a malignant obstruction located in the distal stomach or duodenum and were scheduled for placement of an uncovered self-expandable duodenal stent. Patients who had undergone previous treatment for GOO symptoms (i. e. stent placement or palliative gastrojejunostomy) or had a secondary more distally located stricture were excluded from this study.

### Duodenal stent placement

Duodenal stent placement was performed under fluoroscopic guidance and according to standard procedures. During stent placement, patients were under conscious sedation, propofol sedation, or general anesthesia. Selection of the most appropriate stent design and length was based on the judgement and preference of the endoscopist. Biliary stent placement was performed before duodenal stent placement depending on biliary patency and stricture location.

### Data collection

Endoscopy reports were used to retrieve data regarding the initial duodenal stent placement procedure and subsequent endoscopic procedures, when applicable. These data included the date of procedure, stricture location and length, stent type and measurements, success of stent placement, prior bile drainage, and whether or not the stent was covering the papilla. Data on patient characteristics and procedure-related outcomes, such as age, sex, tumor etiology, prior treatments, the presence of peritoneal deposits and/or ascites, recurrent GOO symptoms after stent placement, AEs, and the cause and time of death, were collected from the electronic patient file. The presence of peritoneal deposits was defined by peritoneal deposits that were described on abdominal imaging and/or where ascites was present.

All patients with recurrent GOO symptoms underwent thorough evaluations to investigate stent patency by imaging techniques and/or upper gastrointestinal (GI) endoscopy. The severity of GOO symptoms were graded according to the Gastric Outlet Obstruction Symptom Score (GOO Symptom Score) 9 . This widely applied scale ranges from zero to three; with a lower score representing more severe symptoms. Every increase of the GOO Symptom Score in the period after stent placement was marked as symptom recurrence. If GOO Symptoms Scores were unavailable, the mention of “re-dysphagia” in the electronic patient file was used as a surrogate marker for GOO symptom recurrence. When an AE occurred during the first 30 days after stent placement, the cause and date of first onset were recorded. The cause and time of death of all patients were collected. If the time of death was unavailable, the municipal registry was consulted.

### Statistical analysis

The primary outcome of this study was GOO symptom-free survival. Secondary outcomes included the AE rate and overall survival. Outcomes were reported as percentages, and means for normally distributed variables or medians for abnormally distributed variables. To compare outcomes for patients treated in different time periods (1998–2009 vs. 2010–2019), the unpaired *t* test, Mann–Whitney *U* test and chi-squared test were used. GOO symptom-free survival and overall survival were compared using the Kaplan–Meier method with log-rank testing. To explore clinical parameters affecting treatment outcomes, binary logistic regression and Cox regression analyses were used. Parameters that were included were selected from previous studies 5 6 7 8 .

All analyses were performed using IBM SPSS statistics, version 25 (IBM Corp., Armonk, New York, USA) and R (R Foundation for Statistical Computing, Vienna, Austria, https://www.R-project.org/). Tests were considered statistically significant if *P* was < 0.05 (two-sided).

## Results

Between January 1998 and November 2019, a total of 187 patients underwent duodenal stent placement at the Erasmus MC University Medical Center. A total of 40 patients were not included for the following reasons: incomplete follow-up after stent placement (n = 21), stricture location other than the distal stomach or duodenum (n = 11), use of a partially covered duodenal stent (n = 4), use of an esophageal stent (n = 1), a previously performed palliative surgical gastrojejunostomy (n = 1), the presence of a second stenosis (n = 1), and a missing endoscopy report (n = 1).

Baseline characteristics of the 147 patients included in this study (62 % men; mean [SD] age 64 12 years) are shown in Table 1 . Pancreatic cancer was the most common cancer type (51 %), followed by cholangiocarcinoma (12 %) and stomach cancer (10 %). A total of 49 patients (33 %) were treated with chemotherapy and/or radiotherapy prior to stent placement and 18 patients (12 %) with concomitant chemoradiotherapy. The percentage of patients treated with prior chemotherapy increased from 25 % in 1998–2009 to 33 % in 2010–2019. Peritoneal deposits were present in 25 % of patients, ascites in 17 % of patients. The proportion of men was statistically significantly higher in patients treated in the period 2010–2019 (1998–2009 vs. 2010–2019, 51 % vs. 72 %; *P* = 0.01). Other baseline characteristics were not statistically significantly different between the two periods.

**Table 1:** Baseline characteristics of the 147 patients with gastric outlet obstruction included in the study.

	Total (n = 147)	1998–2009 (n = 72)	2010–2019 (n = 75)	* P value 1*
Age, mean (SD), years	64 (12)	63 (12)	64 (12)	0.57
Sex, male, n (%)	91 (62)	37 (51)	54 (72)	**0.01**
Tumor etiology, n (%)				0.35
Pancreas	75 (51)	37 (51)	38 (51)	
Bile duct	18 (12)	6 (8)	12 (16)	
Stomach	14 (10)	7 (10)	7 (9)	
Colorectal	6 (4)	4 (6)	2 (3)	
Duodenum	6 (4)	4 (6)	2 (3)	
Gallbladder	6 (4)	1 (1)	5 (7)	
Other 2	22 (15)	13 (18)	9 (12)	
Prior treatment, n (%)				0.51
None	98 (67)	50 (69)	48 (64)	
Prior chemotherapy	43 (29)	18 (25)	25 (33)	0.36
Prior radiotherapy	1 (1)	1 (1)	0	0.31
Prior chemoradiotherapy	5 (3)	3 (4)	2 (3)	0.97
Concomitant chemotherapy, n (%)	18 (12)	7 (10)	11 (15)	0.36
Peritoneal deposits, n (%)	36 (25)	15 (21)	21 (28)	0.31
Ascites, n (%)	25 (17)	11 (15)	14 (19)	0.59

1 1998–2009 vs. 2010–2019.

2 Carcinoma of unknown primary (n = 4), sarcoma (n = 3), urothelial carcinoma (n = 3), breast carcinoma (n = 2), esophageal carcinoma (n = 2), endometrial carcinoma (n = 1), GIST (n = 1), hepatocellular carcinoma (n = 1), lung carcinoma (n = 1), melanoma (n = 1), ovarian cancer (n = 1), renal cell carcinoma (n = 1), Merkel cell carcinoma (n = 1).

### Duodenal stent placement

The median time between initial diagnosis and duodenal stent placement was 3.5 months in 1998–2009 and 6 months in 2010–2019 ( *P* = 0.54). Duodenal stent placement was technically successful in 143 of 147 patients (97 %; 1998–2009 vs. 2010–2019, 94 % vs. 100 %; *P* = 0.04). Reasons for unsuccessful stent placement were inability to pass the guidewire (n = 2), unspecified technical issues (n = 1), and a percutaneous transhepatic drain extending into the duodenum that prevented the stent from deploying (n = 1). Most patients were treated under conscious sedation (1998–2009 vs. 2010–2019, 100 % vs. 94 %; *P* = 0.01), but propofol sedation and general anesthesia were also used in the period 2010–2019.

The WallFlex duodenal stent was the most frequently used stent in the period 1998–2009 (72 %), whereas the Evolution duodenal stent was most frequently used in the period 2010–2019 (91 %; *P* < 0.001) ( Table 2 ). A statistically significantly higher rate of duodenal stents overlapping the papilla was observed in the period 1998–2009 (34 % vs. 17 %; *P* = 0.01). The percentage of patients who underwent drainage of the bile system prior to placement of the duodenal stent was comparable (74 % vs. 77 %; *P* = 0.84).

**Table 2:** Characteristics of 143 patients in whom the duodenal self-expandable metal stent was successfully inserted.

	Total (n = 143)	1998–2009 (n = 68)	2010–2019 (n = 75)	* P value 1*
Sedation, n (%)				** < 0.001**
Conscious sedation	138 (96)	68 (100)	70 (94)	
Propofol	4 (3)	0	4 (5)	
General anesthesia	1 (1)	0	1 (1)	
Stent manufacturer, n (%)				** < 0.001**
Evolution	68 (48)	0	68 (91)	
Niti-S	10 (7)	10 (15)	0	
Shu	1 (1)	1 (2)	0	
UltraFlex	1 (1)	1 (2)	0	
WallFlex	55 (39)	49 (72)	6 (8)	
Unknown	8 (6)	7 (10)	1 (1)	
Location obstruction, n (%) 2				
Distal stomach	32 (22)	14 (21)	18 (24)	0.63
D1	67 (47)	33 (49)	34 (45)	0.70
D2	84 (59)	41 (60)	43 (57)	0.72
D3	31 (22)	17 (25)	14 (19)	0.36
D4	4 (3)	2 (3)	2 (3)	0.92
Extent stricture, n (%)				0.21
Single compartment	73 (51)	31 (46)	42 (56)	
Multiple compartments	70 (49)	37 (54)	33 (44)	
External compression, n (%)	22 (15)	17 (25)	5 (7)	**0.002**
Papilla covered, n (%)	36 (25)	23 (34)	13 (17)	**0.01**

1 1998–2009 vs. 2010–2019.

2 In some patients, multiple compartments were obstructed.

### Recurrent symptoms

**Fig. 1:**
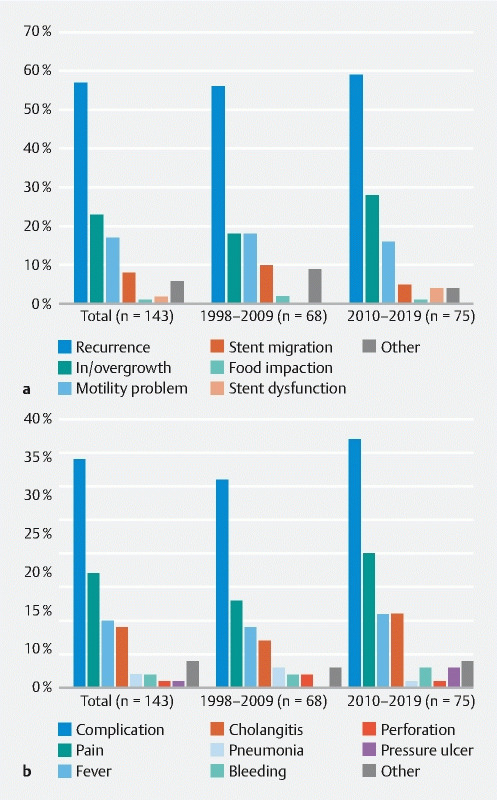
Graphs showing the rates, overall and in the two time periods, for: **a** recurrence of gastric outlet obstruction symptoms, with causes; **b** adverse events, among the 143 patients who underwent successful duodenal stent placement.

During follow-up (median survival time 82 days, range 1–448), a total of 82 patients (57 %) had recurrent GOO symptoms after successful stent placement (1998–2009 vs. 2010–2019, 38 (56 %) vs. 44 (59 %); *P* = 0.74) ( **Table 1 s** , see online-only Supplementary material). The median time until recurrent symptoms was 28 days (1998–2009 vs. 2010–2019, 39 vs. 21 days: *P* = 0.49). The main reasons for recurrent symptoms were tumor ingrowth (23 %), motility problems (17 %, i. e. repeated upper GI endoscopy showed a fully patent stent), and stent migration (8 %) ( Fig. 1a ). An increased rate of stent ingrowth was observed in the period 2010–2019 (18 % vs. 28 %; *P* = 0.14). The stent migration rate decreased over time (10 % vs. 5 %; *P* = 0.27). Other reasons for recurrent GOO symptoms are shown in **Table 1 s** . Motility problems were not significantly different between patients with and without peritoneal deposits (25 % vs. 14 %, respectively; *P* = 0.13).

Multivariable binary logistic regression showed no association between recurrent GOO symptoms and age, sex, prior treatment, chemotherapy, peritoneal deposits and/or ascites, number of strictures (single vs. multiple stenoses), external compression, or period of stent placement ( **Table 2 s** ).

**Fig. 2:**
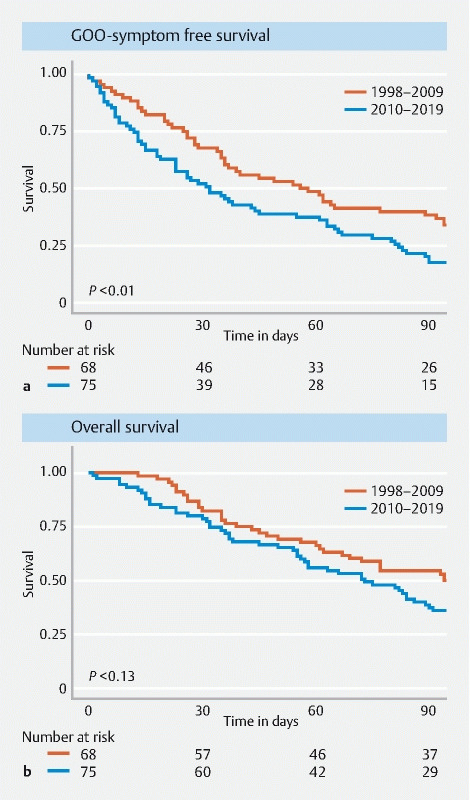
Survival curves comparing patients treated between 1998–2009 and 2010–2019 for: **a** gastric outlet obstruction symptom-free survival; **b** overall survival.

The GOO symptom-free survival was significantly shorter in patients treated in the period 2010–2019 ( *P* = 0.009) ( Fig. 2a ). The 1-, 2– and 3-month GOO symptom-free survival rates were 68 %, 49 %, and 38 %, respectively, between 1998–2009, compared with 52 %, 37 %, and 20 %, respectively, for patients treated between 2010–2019. Multivariable Cox regression analysis showed that the period of stent placement was the only independent predictor of a reduced GOO symptom-free survival (Hazard ratio 1.89; *P* < 0.001) ( Table 3 ).

**Table 3:** Multivariable Cox regression analysis of predictors for shorter gastric outlet obstruction symptom-free survival.

	Hazard ratio	Confidence interval	*P value*
Age	0.99	0.97–1.00	0.06
Sex			0.30
Male	1		
Female	1.21	0.85–1.74	
Prior treatment			0.84
No	1		
Yes	1.04	0.71–1.51	
Chemotherapy			0.11
No	1		
Yes	0.65	0.38–1.10	
Peritoneal deposits and/or ascites			0.07
No	1		
Yes	0.71	0.49–1.03	
Extent of obstruction			0.97
Single compartment	1		
Multiple compartments	1.00	0.71–1.51	
Extrinsic compression			0.36
No	1		
Yes	1.27	0.78–2.11	
Time period			** < 0.001**
1998–2009	1		
2010–2019	1.89	1.31–2.75	

### Adverse events

Of the 143 patients who underwent successful stent placement, 49 (34 %) experienced at least one AE (1998–2009 vs. 2010–2019, 31 % vs. 37 %; *P* = 0.42), after a median time of 4 days ( **Table 1 s** ). The time to occurrence of the first AE did not significantly differ between the two periods (3 vs. 7 days; *P* = 0.14).

The rate of stent-related AEs increased over time, from 31 % in 1998–2009 to 37 % in 2010–2019 ( Fig. 1b ; **Table 1 s** ). Furthermore, when an AE occurred, there was a tendency for multiple AEs to occur in the same patient, especially for patients treated between 2010–2019. This is reflected by the notably higher absolute number of AEs in this period (1998–2009 vs. 2010–2019, 26 vs. 40). The number of major AEs increased from 17 (25 %) to 25 (33 %), and minor AEs from 9 (13 %) to 15 (20 %) in the more recent period.

Major AEs included fever (n = 14), cholangitis (n = 13), hemorrhage (n = 3), pneumonia (n = 3), deep venous thrombosis (n = 3), perforation (n = 2), pressure necrosis (n = 2), delirium (n = 1), and pancreatitis (n = 1). All major AEs increased over time, except pneumonia, which slightly decreased from two events to one. Fistulas were not observed in this study. One patient died owing to a stent-related perforation 1 day after stent placement. Cholangitis was more common in patients in whom the duodenal stent overlapped the papilla (overlap vs. no overlap, n = 5 (14 %) vs. n = 8 (7 %); *P* = 0.23). Pain was the only reported minor AE (n = 24). An increased rate of stent-related pain was observed in patients treated in the period 2010–2019 (13 % vs. 20 %; *P* = 0.28).

Prior treatment with chemotherapy and/or radiotherapy was the only independent risk factor for AEs (OR 2.53; *P* = 0.02) ( Table 4 ). A total of 23 patients who underwent prior treatment (47 %) developed an AE compared with 26 patients with no prior treatment (27 %).

**Table 4:** Multivariable binary logistic regression analysis of risk factors for the occurrence of adverse events.

	Odds ratio	Confidence interval	*P value*
Age	0.99	0.96–1.02	0.48
Sex			0.47
Male	1		
Female	0.75	0.35–1.62	
Prior treatment			**0.02**
No	1		
Yes	2.53	1.17–5.47	
Extent of obstruction			0.32
Single compartment	1		
Multiple compartments	0.69	0.33–1.44	
Time period			0.69
1998–2009	1		
2010–2019	1.16	0.56–2.44	

### Overall survival

At the time of analysis, 142 of 143 patients (99 %) had died, with 141 patients dying because of disease progression and one dying owing to a stent-related AE. Overall survival was lower in patients treated in the more recent period, but this was not statistically significant ( *P* = 0.13) ( Fig. 2b ). The 1-, 2– and 3-month overall survival rates were 84 %, 68 %, and 54 %, respectively, in the period 1998–2009, compared with 80 %, 56 %, and 39 % in the period 2010–2019.

## Discussion

This is the largest study to date to evaluate the clinical outcome of uncovered duodenal stent placement for the palliative treatment of malignant GOO symptoms. This study, including 147 patients over a period of 20 years, provides a valuable insight into time trends in the efficacy and safety of duodenal stent placement. We showed that duodenal stent placement remains a successful treatment option for short-term relief of GOO symptoms. However, the rates of both GOO symptom recurrence and AEs are substantial, with the GOO symptom-free survival in particular decreasing in the last 10 years. Furthermore, prior treatment with chemotherapy and/or radiotherapy was found to be significantly associated with an increased risk of AEs.

Recent changes in treatment strategies have to be taken into account when evaluating the observed significant decrease in GOO symptom-free survival. Since the publication of the SUSTENT trial in the early 2010 s, duodenal stent placement has mainly been recommended for patients with an expected survival of less than 2 months, whereas surgical gastrojejunostomy has been recommended for patients with a life expectancy longer than 2 months 1 . As a result, patients treated with a duodenal stent were likely to have a lower life expectancy in the last 10 years, introducing a considerable inclusion bias for this study. In our study, a lower overall survival in 2010–2019 (although not reaching statistical significance) supports this assumption. This shift in patient selection might explain the decrease in GOO symptom-free survival in the last 10 years. Not only because overall survival has a direct impact on GOO symptom-free survival, but also because it is known that patients with a poor overall clinical condition may have GOO symptoms such as nausea, vomiting and early satiety due to factors other than duodenal stent dysfunction, which can negatively affect a patient’s condition.

In the present study, more than half of the patients (59 %) experienced recurrent GOO symptoms, after a median time of 28 days. This recurrence rate is much higher than has been previously reported for uncovered stents, with prospective studies showing rates ranging from 16 % up to 30 % 7 . In the present study, tumor in- or overgrowth influenced GOO symptom-free survival the most, accounting for 23 % of GOO recurrence. This percentage is comparable to other studies with uncovered duodenal stents, which are prone to tumor ingrowth but are less likely to migrate than covered stents. Although stent occlusion is not a desired outcome, it can be managed with additional stent therapy. Our higher recurrence rate may largely be explained by the fact that also nonstent-related causes of GOO symptoms were included, with motility problems (defined as repeated upper GI endoscopy revealing a patent stent) causing 17 % of our recurrence rate. Other studies did not include this outcome, but we believe it provides clinically relevant information. The follow-up time did not affect the GOO recurrence rates, as our median survival time of 82 days is comparable with other studies.

The presence of peritoneal deposits, Karnofsky performance status, and use of chemotherapy have previously been reported to be related to the clinical success of duodenal stent placement 5 6 7 10 . In our study, additional regression analysis did not identify any clinical parameters related to the recurrence of symptoms. A potential explanation for this difference might be an underestimation of the number of patients with peritoneal deposits in our study, which was considerably lower at 25 % compared with 44 %–56 % in other studies 7 11 12 . Data on Karnofsky performance status was not available owing to the retrospective design of this study.

Not only GOO symptom recurrence but also stent-related AEs have a negative impact on quality of life in patients with incurable disease. In previous studies, the rate of AEs after duodenal stent placement has varied widely, ranging from 5 % up to 42 % 3 5 7 13 14 15 16 17 18 19 20 . In this study, more than one-third of patients experienced at least one AE in the first 30 days after duodenal stent placement. Major iatrogenic complications, such as perforation, bleeding, and pressure ulcers, were rare in both time periods. Perforation rates remained stable over time but there was a slight increase in bleeding and pressure ulcers in more recent years. Furthermore, fever and cholangitis rates were highest in 2010–2019. The increase in cholangitis is remarkable considering the lower rate of duodenal stents overlapping the papilla in this period, which would have been expected to reduce cholangitis rates. A possible explanation for this observation might be found in the etiology of the underlying malignant stricture, with relatively more patients with gallbladder or bile duct cancer in recent years, possibly increasing the risk of cholangitis.

The reason why the number of duodenal stents that covered the papilla was lower in more recent years is not known. Possibly the underlying malignancies, with more biliary malignancies in recent years, may play a role. First, hilar biliary tract malignancies have a tendency to grow more proximally than for example pancreatic tumors, which could result in a more proximal stent placement (i. e. not covering the papilla) in more recent years. Second, most endoscopists will preferably not cover the papilla if gallbladder or bile duct cancer is present, to enable potential endoscopic (re)interventions of the bile duct.

Furthermore, a notable increase of stent-related pain was observed in the last 10 years. More patients were treated with prior chemotherapy and/or radiotherapy in 2010–2019 compared with 1998–2009, which can be proposed as an explanation for the overall increase in both major and minor AEs. Multivariable regression analysis revealed that prior chemotherapy and/or radiotherapy was significantly associated with the risk of AEs: almost half of these patients developed at least one stent-related AE, compared with 27 % of patients with no prior therapy. The association between stent-related AEs and prior treatment has been described in patients undergoing esophageal stent placement 21 , but no studies have explored this association for duodenal stent placement.

Remarkably, pneumonia was the only AE that decreased over time. Unfortunately, pneumonia rates were too low to analyze the relation between the risk of getting pneumonia and altered sedation strategies or the placement of a nasogastric tube prior to duodenal stent placement. In addition, all AE numbers were relatively low in this study; therefore, it is challenging to prove any causal relationship between a parameter and an AE. In order to improve future outcomes, more studies are needed to investigate the impact of different clinical parameters on the risk of stent-related AEs.

Several limitations of this study should be acknowledged, including its retrospective nature and the heterogeneity of the study population, which is inherent to the duodenal stenting population. Because of the retrospective study design, we were unable to re-collect clinical success rates of duodenal stent placement. Unfortunately, owing to the lack of a structured follow-up scheme to evaluate GOO symptoms, the clinical success directly after stent placement was often not reported in the electronic patient file. Given the limited overall survival in this population, the true success of palliation is best measured by the improvement of GOO symptoms and quality of life, rather than by technical success rates.

Furthermore, the study population was too small to perform stratified analyses for different tumor etiologies, or to analyze potential explanatory parameters for separate AEs. In addition, during the study period, different types of duodenal stent were used. To date, however, no comparative study has shown the superiority of one stent manufacturer over another. Finally, we have used two defined time periods as a variable within regression analyses to explore trends over a 20-year period, which prohibits the assessment of fluctuations in smaller time periods.

In conclusion, our study demonstrates that improvement in the outcome of duodenal stent therapy for malignant GOO symptoms is challenging, despite ongoing technical developments. Although successful for short-term palliation of malignant GOO, duodenal stent placement was related to a considerable risk of GOO symptom recurrence and AEs. Recent changes in management strategies, particularly the increased number of patients being pretreated with chemotherapy and/or radiotherapy, are associated with an increase in AEs. More studies are needed to elucidate the influence of different clinical parameters on stent-related outcomes.

Given the decrease of GOO symptom-free survival and the increase in AEs in the last decade, attention should be paid to informing patients about the benefits and potential risks of duodenal stent placement, especially in patients who have received prior chemo- and/or radiotherapy. Nevertheless, considering the lack of generally applicable alternatives, we believe that duodenal stent placement remains the preferred option for the management of GOO symptoms in patients with an expected short-term survival.

## Supplementary material

Supplementary material Zusatzmaterial
