# Supplementary material for: Self-expandable duodenal metal stent placement for the palliation of gastric outlet obstruction over the past 20 years
Source: Endoscopy. 2022 Jun 23;54(12):1139–46. doi: 10.1055/a-1838-5642 (PMC9708383; doi:10.1055/a-1838-5642)
Supplement: Supplementary file 1 — Supplementary material [file 21630supmat_10-1055-a-1838-5642.pdf]

Supplementary material

Self-expandable duodenal metal stent placement for palliation of gastric outlet obstruction over the past 20 years

Agnes N. Reijm, Pauline A. Zellenrath, Ruben D. van der Bogt, Lydi M. J. W. van Driel, Peter D. Siersema, Marco J. Bruno, Manon C. W. Spaander

Table 1s - Recurrence of GOO-symptoms and adverse events in 143 patients.

	**Total (n=143)**	**1998-2009 (n=68)**	**2010-2019 (n=75)**
**Recurrence of symptoms, n (%)**	82 (57)	38 (56)	44 (59)
In/overgrowth	33 (23)	12 (18)	21 (28)
Motility problems[b]	24 (17)	12 (18)	12 (16)
Stent migration	11 (8)	7 (10)	4 (5)
Food impaction	2 (1)	1 (2)	1 (1)
Stent dysfunction[c]	3 (2)	0	3 (4)
Other[d]	9 (6)	6 (9)	3 (4)
**Adverse events per patient, n (%)**	49 (34)	21 (31)	28 (37)
Pain	24 (17)	9 (13)	15 (20)
Cholangitis	13 (9)	5 (7)	8 (11)
Fever	14 (10)	6 (9)	8 (11)
Pneumonia	3 (2)	2 (3)	1 (1)
Bleeding	3 (2)	1 (2)	2 (3)
Perforation	2 (1)	1 (2)	1 (1)
Pressure ulcer	2 (1)	0	2 (3)
Other[e]	5 (4)	2 (3)	3 (4)
**Total adverse events, n[f]**	66	26	40

[a]1998-2009 *vs* 2010-2019
[b]Repeated upper endoscopy revealing a patent stent.
[c] 2x obstruction caused by external compression on duodenal stent; 1x proximal part of patent stent turned to the bulbus wall, disabling food passage.
[d]4x No endoscopy performed due to poor clinical performance status, stent patency unknown; 2x EUS-GJJ due to perforation after initial stent placement; 1x Ileus; 1x blood clot in stent; 1x obstruction caused by stent in common bile duct.
[e]3x deep venous thrombosis, 1x delirium, 1x pancreatitis.
[f]In some patients more than 1 event, therefore no percentage can be given.

Supplementary material

Table 2s – Binary logistic regression analysis for recurrence of symptoms

	**Exp(B)**	**P-value**
**Age**	0.99	0.32
**Gender**		
Male	1	0.87
Female	1.06	
**Prior treatment**		
No	1	0.83
Yes	0.92	
**Adjuvant chemotherapy**		
No	1	0.34
Yes	0.60	
**Peritoneal deposits**		
No	1	0.98
Yes	1.01	
**Extent of obstruction**		
Single compartment	1	0.13
Multiple compartments	0.59	
**Extrinsic compression**		
No	1	0.37
Yes	0.64	
**Time period**		
1998-2009	1	0.92
2010-2019	1.04	
